# DTD: An R Package for Digital Tissue Deconvolution

**DOI:** 10.1089/cmb.2019.0469

**Published:** 2020-03-11

**Authors:** Marian Schön, Jakob Simeth, Paul Heinrich, Franziska Görtler, Stefan Solbrig, Tilo Wettig, Peter J. Oefner, Michael Altenbuchinger, Rainer Spang

**Affiliations:** ^1^ Department of Statistical Bioinformatics, Institute of Functional Genomics, University of Regensburg, Regensburg, Germany.; ^2^ Department of Physics, University of Regensburg, Regensburg, Germany.; ^3^ Institute of Functional Genomics, University of Regensburg, Regensburg, Germany.

**Keywords:** cell-type deconvolution, loss-function learning, model adaptation, R package

## Abstract

**Digital tissue deconvolution (DTD) estimates the cellular composition of a tissue from its bulk gene-expression profile. For this, DTD approximates the bulk as a mixture of cell-specific expression profiles. Different tissues have different cellular compositions, with cells in different activation states, and embedded in different environments. Consequently, DTD can profit from tailoring the deconvolution model to a specific tissue context.**

**Loss-function learning adapts DTD to a specific tissue context, such as the deconvolution of blood, or a specific type of tumor tissue. We provide software for loss-function learning, for its validation and visualization, and for applying the DTD models to new data.**

## 1. Introduction

The cellular composition of a tumor specimen is a prognostic factor (Ansell and Vonderheide, 2013; Junttila and de Sauvage, 2013). Single-cell RNA sequencing (scRNA-Seq; Wu et al., 2013) can be used to assess this composition experimentally. Digital tissue deconvolution (DTD) emerged as a computational alternative (Cobos et al., 2018). It can be applied retrospectively to bulk gene-expression data without experimental costs.

Let *y* be a bulk gene expression profile, *X* a matrix with cell-type specific reference profiles in its columns, and *c* a vector of cellular proportions. DTD reconstructs *c* through *X* and *y* by y=Xc, where different objective functions can be used to approximate *c* (Mohammadi et al., 2017). One of them is the sum of squared residuals, $||y-Xc|{|}_{2}^{2}$, where the residuals are calculated between observed gene expression *y* and its reconstruction *Xc*.

An important observation is that genes can be weighted to obtain better estimates (Görtler et al., 2018). This is particularly the case (1) if not all cells in the bulk are represented in *X*, (2) if we want to estimate contributions from small cell populations, and (3) if we want to disentangle highly similar cell types. Genes weights can be introduced by replacing the residual sum of squares by

(1) $$\arg\;\underset{c}{\min}∥\text{diag}\;(g)\;(y-Xc){∥}_{2}^{2},$$

with a vector *g* of gene weights $g_{i}\geq 0$. A high weight *g_i_* for gene *i* indicates that it is important for deconvolution, whereas a low weight corresponds to a gene that deteriorates the deconvolution. For example, if the expression of a gene in the tissue differs greatly from the corresponding expression in the reference profiles, it cannot be explained by a linear deconvolution approach.

Loss-function learning (Görtler et al., 2018) detects this problem and adapts *g* to a tissue context. It increases deconvolution accuracy, as shown exemplarily for the deconvolution of bulk melanoma specimens (Görtler et al., 2018).

## 2. Methods

Loss-function learning uses training data to optimize DTD models. These training data are bulk gene-expression measurements *Y* (rows: genes/features, columns: measurements) with known cellular compositions *C* (rows: cellular proportions, columns: measurements). The rational behind loss-function learning is to obtain the vector *g* by minimizing a loss function *L* on the training data.

The package DTD incorporates a correlation-based loss function:

(2) $$\begin{array}{c} L=-\overset{q}{\underset{j=1}{\sum }}\;\text{cor}\;(C_{j,.},{\hat{C}}_{j,.}(g)),\\ \text{subject}\;\text{to}\;g_{i}\geq 0\;\text{and}∥g{∥}_{2}\;=\;1\; \end{array}$$

Here, Ĉ(g) is the solution of Equation (1). The optimization problem is stated in a closed analytical form and is implemented efficiently in C++ for optimal performance.

## 3. Application

### 3.1. Basic function calls

The R package DTD has the basic function call

(3) train_deconvolution_model(X.matrix = X , train.data.list = train.data, …) 

**FIG. 1.**
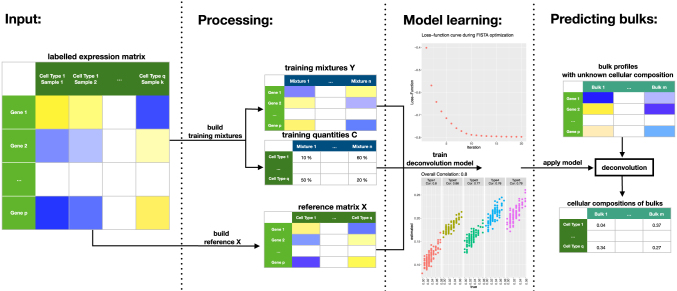
Workflow of a digital tissue deconvolution (DTD) analysis with loss-function learning. Input: an expression matrix, where each sample is labeled with its cell type. Processing: the labeled samples are used to build both a reference matrix and artificial mixtures of known cellular composition. Model learning: the algorithm iteratively searches for parameters *g*, which maximize the correlation between the estimated and the true cellular compositions, where the training data and the reference matrix are used. Here, functions visualize the result and assess the performance of the DTD model. Predicting bulks: the DTD model is applied to bulk gene expression data to estimate the underlying cellular composition.

Here, *X* is a matrix with reference profiles in its columns, and train.data is a list containing “mixtures” *Y* and “quantities” *C*, defined as earlier. train_deconvolution_model returns a deconvolution model that can be applied through the function estimate_c(DTD.model = trained.model, new.data = y) on new data *y*, which is a vector or matrix with bulk gene-expression levels. The function estimate_c returns estimated cellular proportions for the bulk profiles *y*. The workflow is summarized in Figure 1.

### 3.2. Generate artificial training data from scRNA-Seq data

Loss-function learning requires training data. These data can be generated experimentally, for example, through fluorescence-activated cell sorting (FACS)-based cell counting combined with bulk RNA sequencing. Alternatively, these data can be generated artificially from scRNA-Seq experiments. For this purpose, we implemented functions that automatically generate training mixtures *Y* and their corresponding compositions *C*. An example for such a function call is mix_samples(exp.data = sc.counts, pheno = sc.pheno, …). This function uses, for example, raw counts from scRNA-Seq (sc.counts), where columns correspond to cells and rows to genes. Furthermore, the vector sc.pheno ascribes cell types to the respective columns in sc.counts. The function returns a list with the components *Y* and *C*, which correspond to artificial mixtures and their underlying cellular compositions, respectively. A similar function is provided to generate a reference matrix *X*.

## 4. Summary

We provide software for the digital deconvolution of bulk gene-expression data. We take into account that DTD needs to be adapted to a specific type of tissue. It is not likely that there is a universal deconvolution formula, which performs consistently well in all settings. The most reliable results might be obtained by optimized models that have been trained within a machine learning framework.

In this study, we provide software for such a framework, called loss-function learning, and for the application of the learned deconvolution models to bulk data. It is computationally tractable and easy to use.

A DTD tutorial is available as Supplementary Data. There, we give a comprehensive example that shows how data need to be processed and how results can be visualized.

In summary, we provide the R package DTD that contains software to systematically improve the performance of DTD algorithms.

## Supplementary Material

Supplemental data

## Availability and Implementation

DTD is available under https://github.com/MarianSchoen/DTD.
