# Supplementary material for: DTD: An R Package for Digital Tissue Deconvolution
Source: J Comput Biol. 2020 Mar 11;27(3):386–9. doi: 10.1089/cmb.2019.0469 (PMC7074920; doi:10.1089/cmb.2019.0469)
Supplement: Supplemental data [file Supp_Data.pdf]

DTD
Tutorial

Marian Schön[1], Jakob Simeth[1], Paul Heinrich[1], Franziska Görtler[1], Stefan Solbrig[2], Tilo Wettig[2], Peter J. Oefner[3], Michael Altenbuchinger[1], Rainer Spang[1]

marian.schoen@klinik.uni-regensburg.de

[1] *Statistical Bioinformatics, Institute of Functional Genomics, University of Regensburg, Germany*
[2] *Department of Physics, University of Regensburg, Germany*
[3] *Institute of Functional Genomics, University of Regensburg, Germany*

2019-12-03

Contents

Overview **1**

Introduction to DTD **2**

Data **2**
Downloading 2
Read in and preprocess 3
Pheno information 3
Single-Cell Profiles 4
Reconstruct inferred bulk profiles 4

DTD Analysis **6**
Generate reference matrix X 6
Generate training and test 'in-silicio' mixtures 7
Assess the baseline deconvolution model 8
Train a deconvolution model 10
Assess the trained deconvolution model 10
Deconvolute bulk profiles 17

References **19**

Overview

Loss-Function Learning (Görtler et al. 2018) allows to adapt a deconvolution model to a specific tissue context. Here, we show an exemplary analysis using the R package *DTD* (Digital Tissue Deconvolution), https://github.com/MarianSchoen/DTD,
and demonstrate how loss-function learning increases the deconvolution accuracy.

```
library(DTD)
# for downloading an exemplary scRNA-Seq data set form ncbi:
library(GEOquery)
# in order to show the runtime of our optimization:
library(tictoc)
```

A complete *DTD* analysis consists of the following steps:

1. Preprocess labelled expression profiles (e.g. scRNA-Seq)
2. Generate a reference matrix and 'in-silicio' mixtures
3. Train the model
4. Assess the quality of the model
5. Deconvolute bulks to reconstruct their cellular composition

Introduction to DTD

The bulk gene expression profile of a tissue combines the expression profiles of all cells in this tissue. Digital tissue deconvolution (*DTD*) addresses the inverse problem: Given the expression profile y of a tissue, what is the cellular composition c of cells X in that tissue? The cellular composition c can be estimated by

$$\arg\min_{c} ||y - Xc||_2^2.$$

This can be generalized by introducing a vector g

$$\arg\min_{c} ||\text{diag}(g)(y - Xc)||_2^2. \qquad (2)$$

Every entry g_i of g reflects importance of gene i for the deconvolution process. It can either be prior knowledge, or learned on training data. Training data consists of artificial bulk profiles Y and their corresponding cellular compositions C. We generate artificial mixtures Y and their quantities C with single-cell RNA-Seq profiles. The underlying idea of loss-function learning *DTD* is to obtain the vector g by minimizing a loss function L on the training set:

$$L = -\sum_{j} \text{cor}(C_{j,.}, \widehat{C}_{j,.}(g)),$$

$$\text{subject to } g_i \geq 0 \text{ and } ||g||_2 = 1.$$

Here, $\widehat{C}_{j,.}(g)$ is the solution of formula (2). During training we iteratively adjust the g vector in the direction of the gradient ∇L. The resulting g vector leads to cellular estimates $\widehat{C}(g)$ that correlate best with the known estimates C.

Data

DTD provides an algorithm to adapt a deconvolution model to its tissue context. For this, it requires labelled data. The following sections show how to automatically download and process one data set, published by (Tirosh et al. 2016). However, other scRNA-Seq data sets might be stored in different formats and, therefore, need different processing scripts. Our R-package *DTD* focuses only on training a deconvolution model, not on the preprocessing.
In this exemplary analysis we adapt a deconvolution model to a single-cell RNA-Seq data set of melanomas (Tirosh et al. 2016). The data set can be downloaded via GEO entry 'GSE72056':

Downloading

Here, we download the complete supplement file, because it includes gene counts and cell labels (tumor, cell type).

```
# download the supplemental file:
raw <- getGEOSuppFiles(
  GEO = "GSE72056"
)
# the getGEOSuppFiles function creates a directory named "GSE72056",
```

```r
# in which the .txt.gz is stored. Read it in via:
tirosh.melanoma <- read.table(
  file = "GSE72056/GSE72056_melanoma_single_cell_revised_v2.txt.gz",
  stringsAsFactors = FALSE,
  header = TRUE,
  sep = "\t"
)
```

Read in and preprocess

Notice, that in the `tirosh.melanoma` object, the pheno information (tumor, malignant cell, and non-malignant cell type) is combined with the count matrix.
We split `tirosh.melanoma` into a `tm.pheno` (tm for tirosh melanoma) and `tm.expr` object, respectively. In the data set, the count matrix is stored as log2 values. *DTD* reconstructs bulks as a weighted sum of cell profiles, therefore, the data must be stored in linear space.

```r
# The first 3 rows hold:
#   - "tumor"
#   - "malignant(1=no,2=yes,0=unresolved)",
#   - "non-malignant cell type (1=T,2=B,3=Macro.4=Endo.,5=CAF;6=NK)")
tm.pheno <- as.matrix(tirosh.melanoma[1:3, -1])
rownames(tm.pheno) <- tirosh.melanoma[1:3, 1]

# workaround for the duplicated rownames:
row.names <- as.character(tirosh.melanoma[4:nrow(tirosh.melanoma), 1])
dupls.pos <- which(duplicated(row.names))
unique.names <- paste0(row.names[dupls.pos], "--2")
row.names[dupls.pos] <- unique.names
# undo log transformation from tirosh (DTD works on an additive scale, not a multiplicative) ...
tm.expr <- as.matrix(2^(tirosh.melanoma[4:nrow(tirosh.melanoma), -1]) - 1)
# ... normalize each profile to a fixed number of counts...
tm.expr <- normalize_to_count(tm.expr)
# ... and reset the rownames.
rownames(tm.expr) <- row.names
```

Pheno information

The `tm.pheno` matrix holds 3 rows. The first row indicates the `"tumor"`. Every single-cell profile with the same `"tumor"` entry originates from the same sample. The second row holds the `"malignant"` information. The third row gives the annotated cell type for each profile. All pheno entries are adapted from (Tirosh et al. 2016). In the raw data, `"malignant"` and `"CellType"` are given as numeric values. The following functions map these numeric values to strings:

```r
map.malignant <- function(x) {
  if (x == 1) return("NOT_malignant")
  if (x == 2) return("malignant")
  if (x == 3) return("unresolved")
  return("unassigned")
}
map.cell.type <- function(x) {
  if (x == 1) return("T")
  if (x == 2) return("B")
  if (x == 3) return("Macro")
  if (x == 4) return("Endo")
```

```
  if (x == 5) return("CAF")
  if (x == 6) return("NK")
  return("unknown")
}
tm.pheno.readable <- data.frame(
  "tumor" = tm.pheno["tumor", ],
  "malignant" = sapply(
    tm.pheno["malignant(1=no,2=yes,0=unresolved)", ],
    map.malignant
  ),
  "CellType" = sapply(
    tm.pheno["non-malignant cell type (1=T,2=B,3=Macro.4=Endo.,5=CAF;6=NK)", ],
    map.cell.type
  )
)
# remove 'tm.pheno', only use 'tm.pheno.readable
rm(tm.pheno)
```

Single-Cell Profiles

In the data set there are 23686 features (rows), and 4645 single-cell profiles (columns). Each profile is normalized to a fixed number of counts (via the `DTD::normalize_to_count` function, in the preprocess section):

```
head(apply(tm.expr, 2, sum))
```

```
##      Cy72_CD45_H02_S758_comb    CY58_1_CD45_B02_S974_comb
##                        23686                        23686
##      Cy71_CD45_D08_S524_comb  Cy81_FNA_CD45_B01_S301_comb
##                        23686                        23686
##   Cy80_II_CD45_B07_S883_comb Cy81_Bulk_CD45_B10_S118_comb
##                        23686                        23686
```

Notice, that each row in the `tm.expr` matrix corresponds to a feature, and a column to a single-cell profile:

```
tm.expr[1:5, 1:2]
```

```
##          Cy72_CD45_H02_S758_comb CY58_1_CD45_B02_S974_comb
## C9orf152                  0.0000                   0.00000
## RPS11                   140.7362                  78.37141
## ELMO2                     0.0000                   0.00000
## CREB3L1                   0.0000                   0.00000
## PNMA1                     0.0000                   0.00000
```

Reconstruct inferred bulk profiles

The data set consists of 4645 single-cell profiles from 19 tumors. In order to apply the model to bulk data, we reconstruct the bulk profiles by summing up all single-cell profiles from the same tumor.

```
tumor.names <- as.character(unique(tm.pheno.readable$tumor))

# initialize emtpy expression matrix ...
bulk.exprs <- matrix(NA,
  nrow = nrow(tm.expr),
  ncol = length(tumor.names)
)
```

```
rownames(bulk.exprs) <- rownames(tm.expr)
colnames(bulk.exprs) <- tumor.names

# ... and pheno matrix
bulk.pheno <- matrix(0,
  nrow = length(tumor.names),
  ncol = length(unique(tm.pheno.readable$CellType))
)
rownames(bulk.pheno) <- tumor.names
colnames(bulk.pheno) <- unique(tm.pheno.readable$CellType)

# iterate over each tumor, and sum up all its profiles:
for (l.tumor in tumor.names) {
  tmp.samples <- which(tm.pheno.readable$tumor == l.tumor)
  bulk.exprs[, l.tumor] <- rowSums(tm.expr[, tmp.samples])

  tmp.table <- table(tm.pheno.readable[tmp.samples, "CellType"])
  bulk.pheno[l.tumor, names(tmp.table)] <- tmp.table / sum(tmp.table)
}
# normalize the profiles:
bulk.exprs <- normalize_to_count(bulk.exprs)
```

We split the scRNA-Seq profiles into a training and test set based on the `"tumor"`. For reproducibility, we report test and training tumors.

```
# split the melanoma data into a test and training set
# (scRNA-Seq profiles of a melanoma are either in the
# training, or in the test set)
set.seed(20)
train.pos <- sample(
  x = 1:length(tumor.names)
  , size = 0.5 * length(tumor.names)
  , replace = FALSE
  )
test.pos <- (1:length(tumor.names))[-train.pos]

cat("Training tumors: ", tumor.names[train.pos])
```

```
## Training tumors:  89 84 80 53 60 75 58 72 81
```

```
cat("Test tumors: ", tumor.names[test.pos])
```

```
## Test tumors:  71 74 79 82 59 67 65 78 88 94
```

```
train.melanomas <- tumor.names[train.pos]
test.melanomas <- tumor.names[test.pos]

train.profiles.pos <- which(tm.pheno.readable$tumor %in% train.melanomas)
train.pheno <- tm.pheno.readable[train.profiles.pos, ]
# notice, tm.expr and tm.pheno.readable are in the same order
# => therefore, subsetting by position in both objects is fine
train.profiles <- tm.expr[, train.profiles.pos]

test.profiles.pos <- which(tm.pheno.readable$tumor %in% test.melanomas)
test.pheno <- tm.pheno.readable[test.profiles.pos, ]
test.profiles <- tm.expr[, test.profiles.pos]
```

DTD Analysis

All previous steps address the downloading and processing of exemplary data set. In this section, the *DTD* analysis starts, all function calls are data-set independent.
Start your *DTD* analysis by constructing a vector that maps profiles to cell types, and choose which cell types should be included in the reference matrix X. In the training data (and the bulk data) there might be cell types that are not in the reference matrix, yet, the *DTD* algorithm finds the optimal g-vector to deconvolute the present cell types. The effect can be seen by setting `include.in.X` to, e.g., `c("B", "T")` in the following code chunk.

```
indicator.vector <- as.character(tm.pheno.readable$CellType)
names(indicator.vector) <- rownames(tm.pheno.readable)
indicator.train <- indicator.vector[train.profiles.pos]
indicator.test <- indicator.vector[test.profiles.pos]
include.in.X <- c("B", "CAF", "Macro", "NK", "T")

print(head(indicator.vector))
```

```
##      Cy72_CD45_H02_S758_comb    CY58_1_CD45_B02_S974_comb
##                          "B"                          "T"
##      Cy71_CD45_D08_S524_comb  Cy81_FNA_CD45_B01_S301_comb
##                    "unknown"                    "unknown"
##   Cy80_II_CD45_B07_S883_comb Cy81_Bulk_CD45_B10_S118_comb
##                    "unknown"                    "unknown"
```

```
print(head(indicator.vector))
```

```
##      Cy72_CD45_H02_S758_comb    CY58_1_CD45_B02_S974_comb
##                          "B"                          "T"
##      Cy71_CD45_D08_S524_comb  Cy81_FNA_CD45_B01_S301_comb
##                    "unknown"                    "unknown"
##   Cy80_II_CD45_B07_S883_comb Cy81_Bulk_CD45_B10_S118_comb
##                    "unknown"                    "unknown"
```

Generate reference matrix X

Using the `indicator.vector` and the `include.in.X` vectors we can generate a reference matrix X. Here, for every cell type in `include.in.X` we randomly select 20% of all cells of that type, and average them. All samples that have been used in creating X must not be used any further, and have been excluded from the expression matrix.

```
# The 'sample_random_X' generates a reference matrix X,
# by randomly selecting 'percentage.of.all.cells' per cell type,
# and averaging over them.
sample.X <- sample_random_X(
  included.in.X = include.in.X,
  pheno = indicator.train,
  expr.data = train.profiles,
  percentage.of.all.cells = 0.2,
  normalize.to.count = TRUE
)
# the function returns the 'X.matrix' ...
X.matrix <- sample.X$X.matrix
```

```
# .. and all profiles that have been used to generate X.
# The already used profiles must not be used any further.
samples.to.remove <- sample.X$samples.to.remove

# Hence, they must be removed from the 'train.profiles' ...
remaining.train.profiles <- train.profiles[ ,
  -which(colnames(train.profiles) %in% samples.to.remove)]
# ... and the corresponding indicator vector.
remaining.indicator.train <- indicator.train[colnames(remaining.train.profiles)]
```

Next, we reduce the number of features. Loss-function learning digital tissue deconvolution performs a feature selection, and we could start the algorithm on all 23686 features. In this example, due to run time, we preselect a set of features. The preselection is done via standard deviation in the reference matrix X.

```
n.features <- 500

# calculate standard deviation per feature in X:
sds.in.x <- rowSds(X.matrix)
names(sds.in.x) <- rownames(X.matrix)
sorted.sds.in.x <- sort(sds.in.x, decreasing = TRUE)
# and select the top 'n.features':
selected.features <- names(sorted.sds.in.x)[1:n.features]

# reduce the feature set for all expression matrices:
X.matrix <- X.matrix[selected.features, ]
remaining.train.profiles <- remaining.train.profiles[selected.features, ]
test.profiles <- test.profiles[selected.features, ]
bulk.exprs <- bulk.exprs[selected.features, ]
```

Generate training and test 'in-silicio' mixtures

Next, we randomly mix single-cell profiles, resulting in artificial 'in-silicio' bulks. We use the training set to train the model, and after that validate it on a test set. To this end, split all remaining profiles in a disjoint training and test set. Mix the sets with the *DTD* function `mix_samples`. The number of artificial bulk samples can be set as an hyperparameter. The runtime of the optimization increases linear with the number of training samples and the number of features. Another hyperparameter is the number of single-cell profiles per 'in-silicio' mixture. This parameter depends on the dimension of the data set.

```
# rule of thumb:
n.samples <- n.features
# there are ~2000 SC profiles in the training set,
# choose 'n.per.mixture' ~20% of that => 400
n.per.mixture <- 400

training.data <- mix_samples(
  expr.data = remaining.train.profiles,
  pheno = remaining.indicator.train,
  included.in.X = include.in.X,
  n.samples = n.samples,
  n.per.mixture = n.per.mixture,
  verbose = FALSE
)

# generate test mixtures, using the test profiles
test.data <- mix_samples(
```

```r
  expr.data = test.profiles,
  pheno = indicator.test,
  included.in.X = include.in.X,
  n.samples = n.samples,
  n.per.mixture = n.per.mixture,
  verbose = FALSE
)
```

Assess the baseline deconvolution model

In this section, we show why adapting the deconvolution model to the tissue scenario is important. We deconvolute the artificial test mixtures and the reconstructed bulk profiles with the baseline deconvolution model. If $g_i = 1$ for all genes i, the baseline deconvolution model can be seen in formula (2) - section 'Introduction to *DTD*'. The following pictures show the deconvolution result per cell type for the baseline model. For both, test set and bulk profiles, the deconvolution accuracy is low. On the y axis of the plot the estimated cellular proportions are displayed, on the x axis the true proportions. We report a correlation per cell type, and an overall correlation. The overall correlation averages cell type specific correlations. The second plot shows the deconvolution results on the reconstructed bulks. Here, the training bulks are shown as triangles and the test bulks as circles.

```r
baseline.model <- rep(1, n.features)
names(baseline.model) <- selected.features
untrained.artificial.mixtures.pic <- ggplot_true_vs_esti(
  DTD.model = baseline.model,
  X.matrix = X.matrix,
  test.data = test.data,
  estimate.c.type = "direct",
  title = " test bulks; baseline model: g=1"
)
print(untrained.artificial.mixtures.pic[[1]])
# Here, we only show the first picture of the 'ggplot_true_vs_esti' plot list,
# which is the plot gathering all cell types.
# All following entries of 'untrained.art.mixtures.pic' hold a
# true vs esti plot with only one cell type.

bulk.list <- list(
  "mixtures" = bulk.exprs[selected.features, ],
  "quantities" = t(bulk.pheno[, colnames(X.matrix)])
)
untrained.bulk.deconvolution.pic <- ggplot_true_vs_esti(
  DTD.model = baseline.model,
  X.matrix = X.matrix,
  test.data = bulk.list,
  title = " reconstructed bulks; baseline model: g=1",
  estimate.c.type = "direct",
  shape.indi = ifelse(tumor.names %in% train.melanomas, "train", "test"),
  show.legend = TRUE
)
print(untrained.bulk.deconvolution.pic[[1]])
```

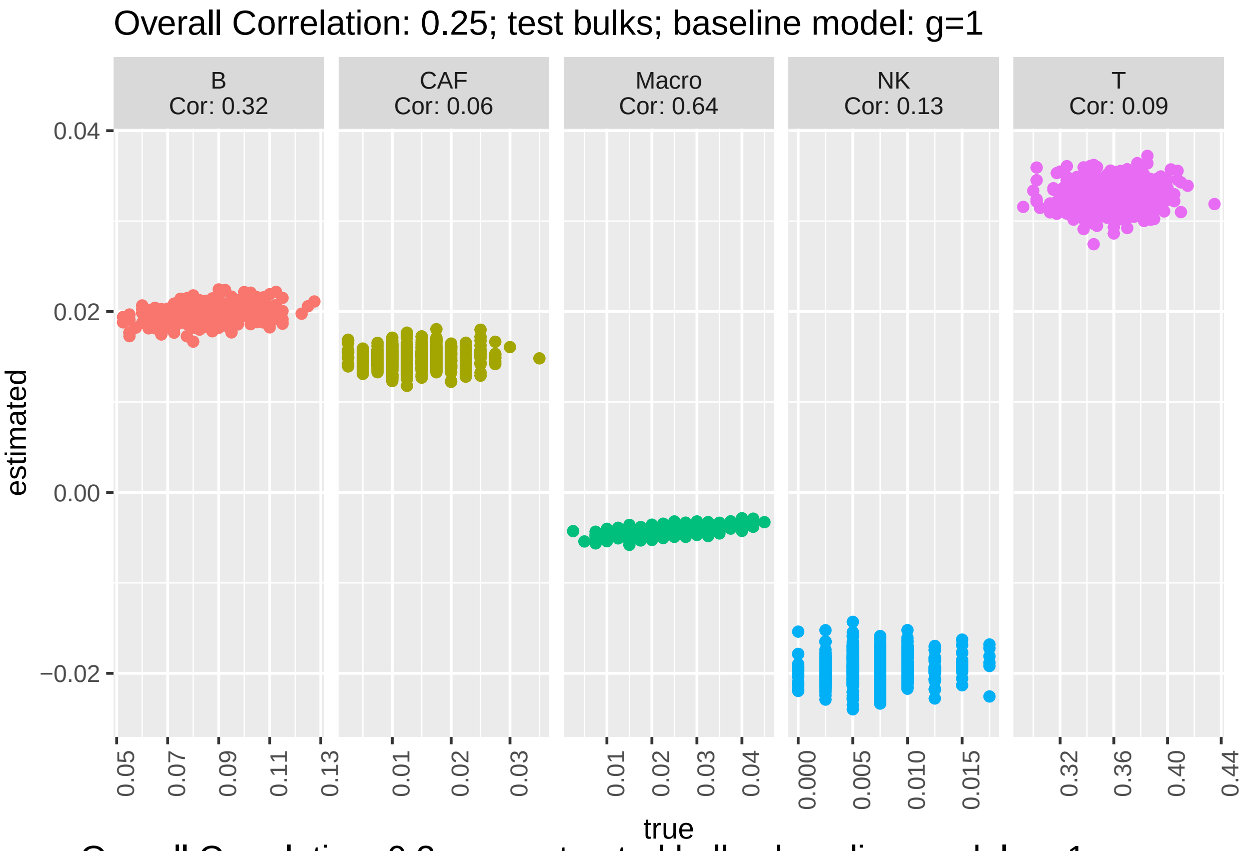
Overall Correlation: 0.25; test bulks; baseline model: g=1
B
Cor: 0.32
CAF
Cor: 0.06
Macro
Cor: 0.64
NK
Cor: 0.13
T
Cor: 0.09
estimated
true

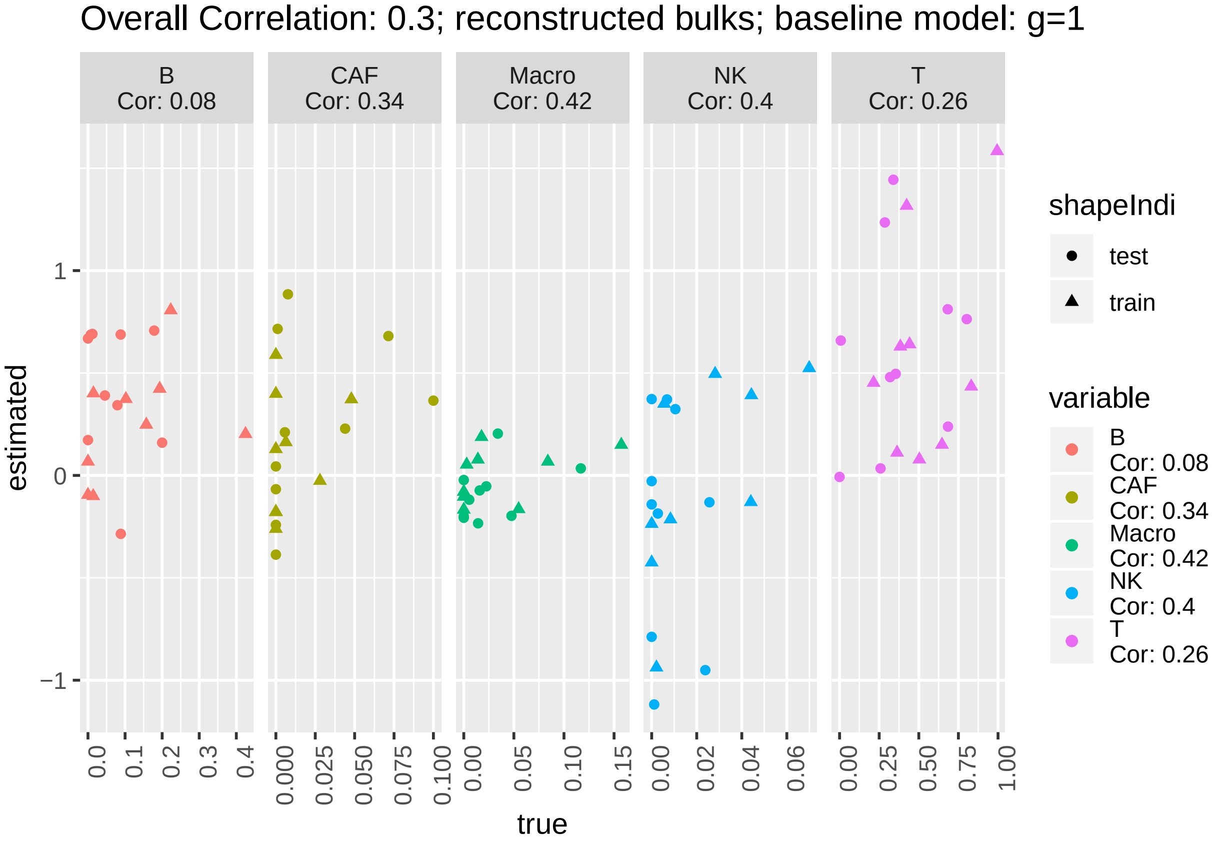
Overall Correlation: 0.3; reconstructed bulks; baseline model: g=1
B
Cor: 0.08
CAF
Cor: 0.34
Macro
Cor: 0.42
NK
Cor: 0.4
T
Cor: 0.26
estimated
true
shapeIndi
test
train
variable
B
Cor: 0.08
CAF
Cor: 0.34
Macro
Cor: 0.42
NK
Cor: 0.4
T
Cor: 0.26

Train a deconvolution model

Using the `train_deconvolution_model` function a deconvolution model is adapted to the tissue. Hyperparameters can be set via `'...'`.
The `train_deconvolution_model` function adapts a DTD model to the training data, and afterwards automatically calls plot functions with default parameters, and stores the pictures in the `pics` entry of the model list. We show the time consumption of our optimization using the R-package `tictoc`, even though the runtime varies between workstations.

```
start.tweak <- rep(1, n.features)
names(start.tweak) <- selected.features
tic("time consumption of optimization: ")
model <- train_deconvolution_model(
  tweak = start.tweak,
  X.matrix = X.matrix,
  train.data.list = training.data,
  test.data.list = test.data,
  estimate.c.type = "direct",
  use.implementation = "cpp"
  )
toc()
```

```
## time consumption of optimization: : 1.026 sec elapsed
```

Assess the trained deconvolution model

In this section, we show visualizations of the training process of the model.

Optimization convergence

In the `convergence` entry, the visualization of the loss L against the iteration of the gradient descent is shown. If the `test.data` is provided to the `ggplot_convergence` function, and all intermediate g vectors are stored in the model, the test convergence is visualized as well. Notice, the optimization procedure uses only the training data, and does not see the test data at any point. Therefore, a temporarily increasing loss on the test data might occur.

```
print(model$pics$convergence)
```

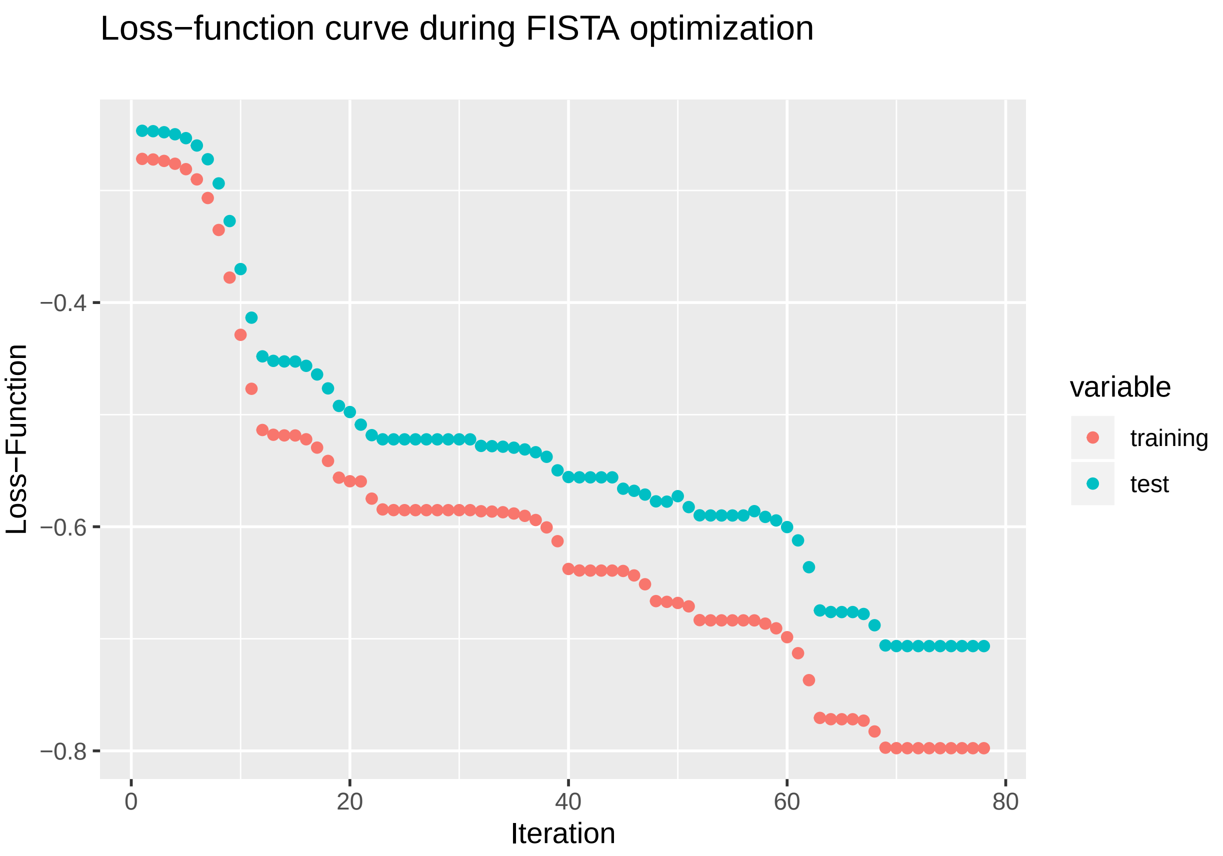
Loss−function curve during FISTA optimization
Loss−Function
−0.4
−0.6
−0.8
0
20
40
60
80
Iteration
variable
training
test

Regression path

In the `path` entry, the regression path of each g_i is visualized over all iterations. Each line in the plot corresponds to one gene. For gene i its line tracks g_i over all iterations. On the y axis, the log10(g + 1) value is shown. Notice, that by default, the legend is not plotted. In the `ggplot_gpath` function the parameter `show.legend` can be set `TRUE`, then a legend is plotted as well.

```
print(model$pics$path)
```

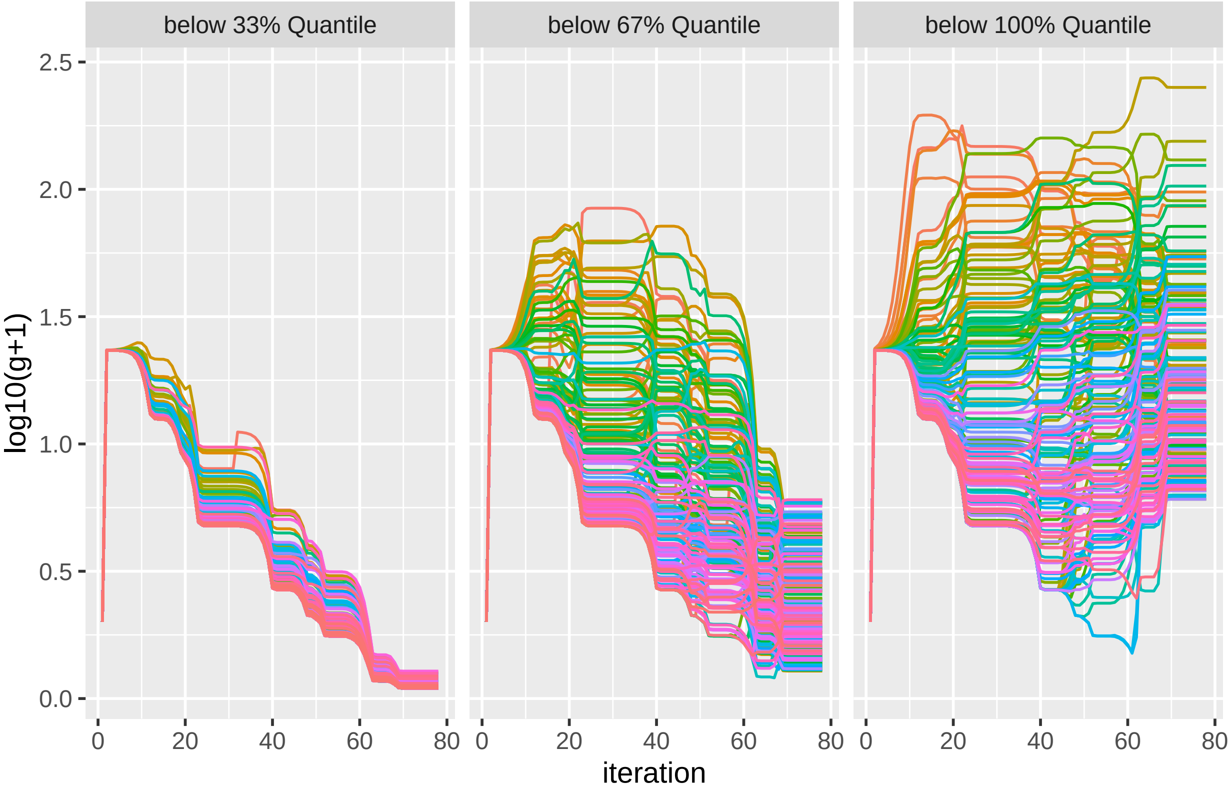


```
path.with.legend <- ggplot_gpath(
  DTD.model = model,
  number.pics = 1,
  subset = c("IGFBP7", "LYZ", "CTSW"),
  show.legend = TRUE
)
print(path.with.legend$gPath)
plot(path.with.legend$legend)
```

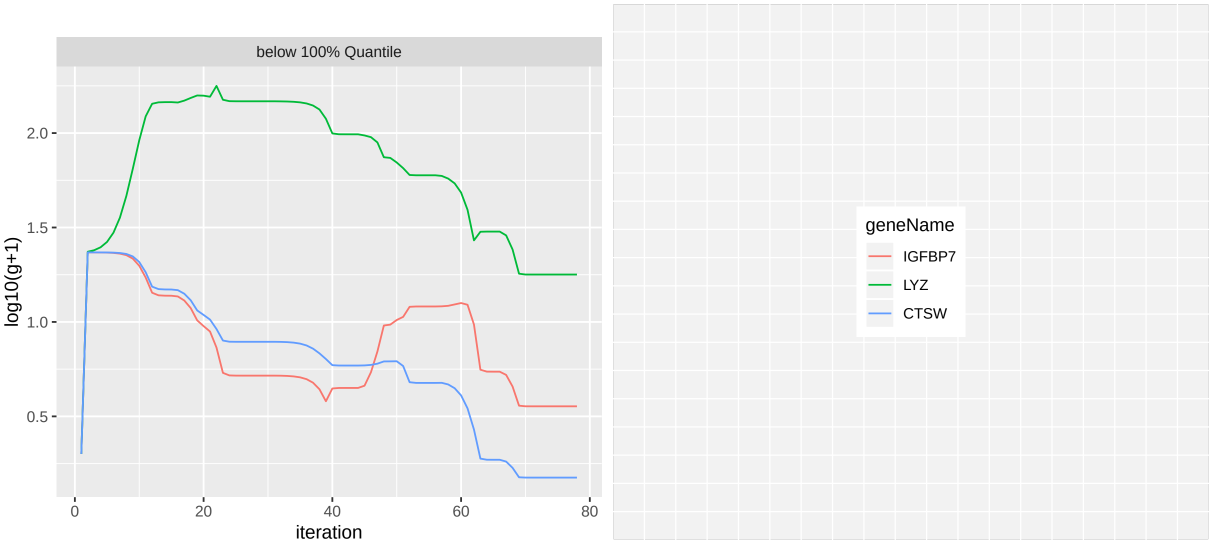
below 100% Quantile
log10(g+1)
2.0
1.5
1.0
0.5
0
20
40
60
80
iteration
geneName
IGFBP7
LYZ
CTSW

Distribution of g

The distribution of the g vector is visualized as a histogram in the `histogram` entry. Remark, by default, we fix $||g||_2 = \#g$, the number of features in g. If gene i is useful for deconvolution, its entry g_i is high. As in the plot before, we visualize log10(g + 1) instead of g.

```
print(model$pics$histogram)
```

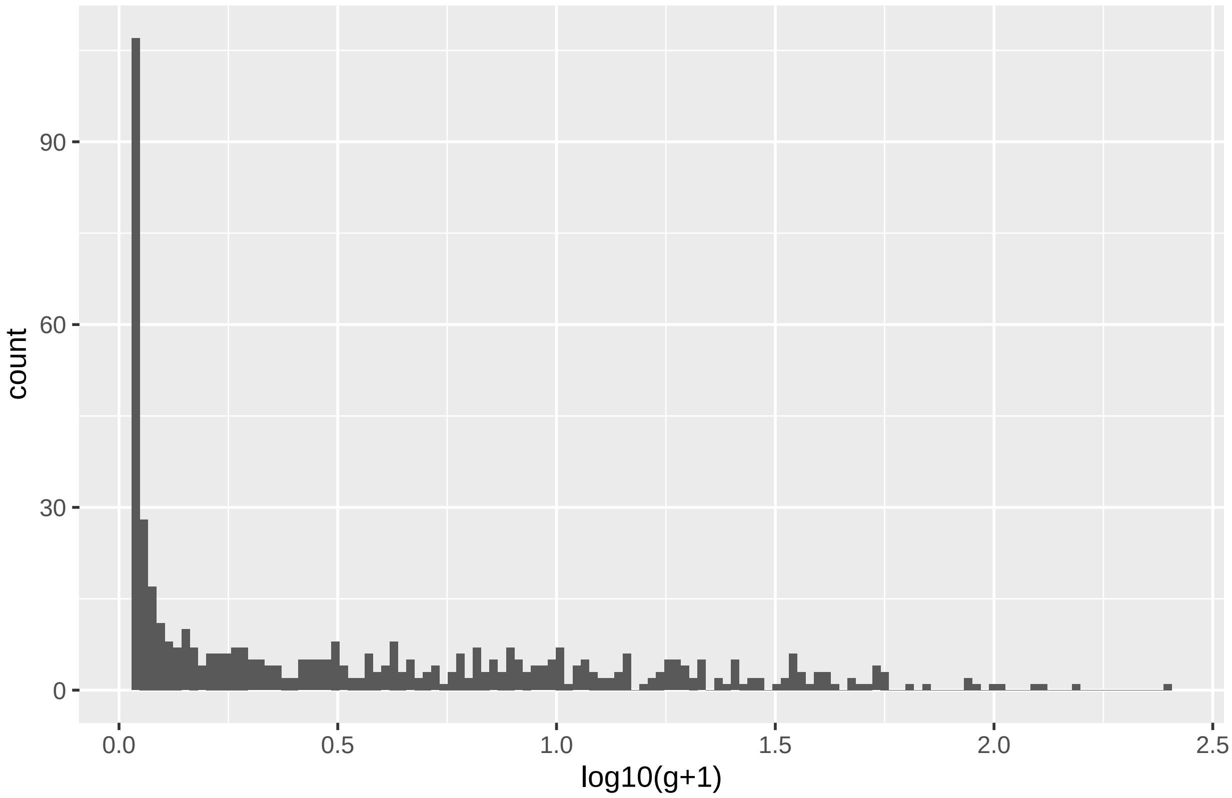

Heatmap of X diag(g) x X

The effect of the g-vector on deconvolution can be visualized when plotting the reference matrix X, weighted by g, as a heatmap. Plot it via the `Xheatmap` entry, or the `ggplot_heatmap` function. Rows and columns are clustered hierarchically. The heatmap becomes more informative if only a subset of features is included. A subset can be selected via the `feature.subset` parameter. If a vector of feature names is provided, only these features are visualized. Alternatively, a number can be provided. Then, the `ggplot_heatmap` function detects features that are important for deconvolution. This is done by iteratively removing one feature from the trained model. Removing a feature, changes the deconvolutoin accuracy. The more important a removed feature was, the lower the deconvolution accuracy gets. Based on this score, the `ggplot_heatmap` function can visualize the most important deconvolution features. In the heatmap, bright yellow fields of the heatmap show that the gene was highly expressed in this cell type. Notice, by default, the log2(expr + 1) is visualized. Using this information, the algorithm can detect marker genes of cell types.

```
#print(model$pics$Xheatmap)
ggplot_heatmap(
  DTD.model = model,
  X.matrix = X.matrix,
  test.data = test.data,
  estimate.c.type = "direct",
  title = "Heatmap of diag(g) x X",
  feature.subset = 20
)
```

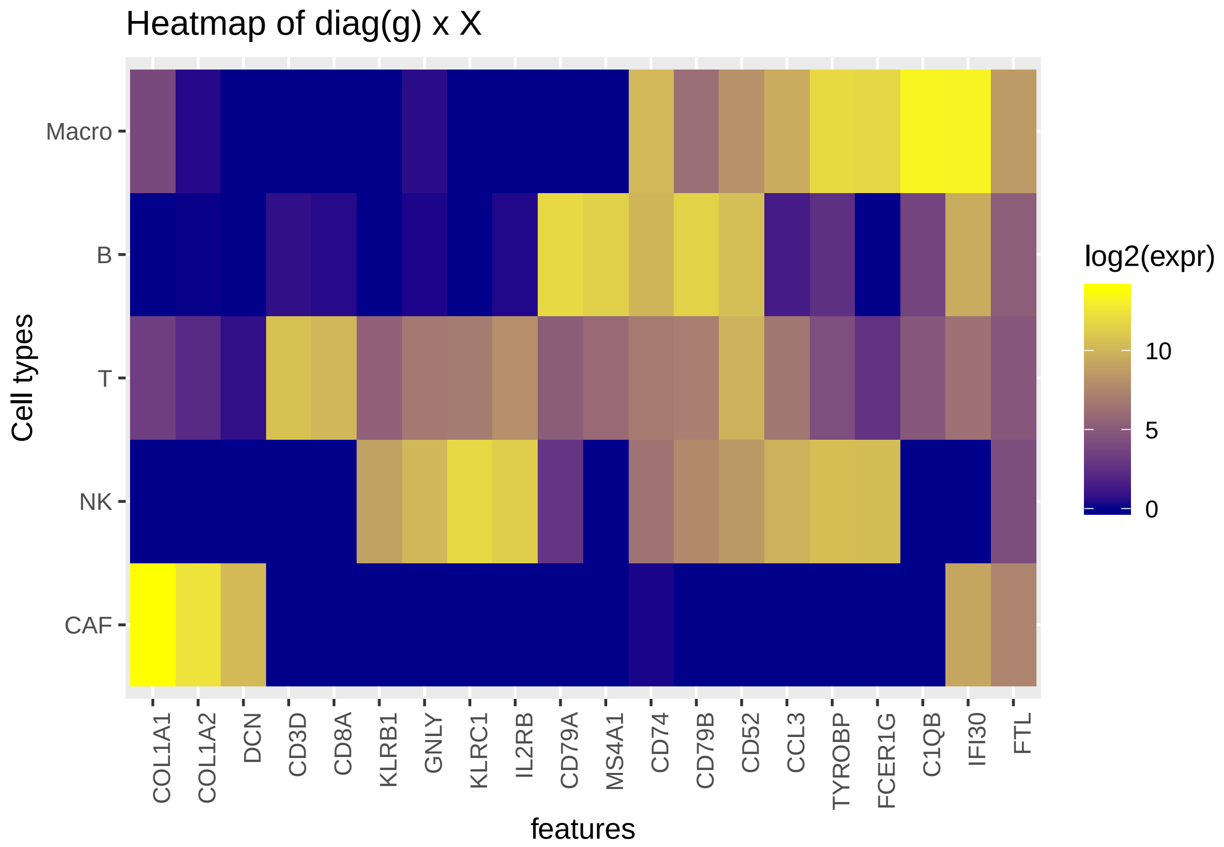

True vs estimated proportions

The 'true C versus estimated $\widehat{C}(g)$' plot (as for the untrained model in section 'Assess the baseline deconvolution model') is plotted. In the cell proportion estimates $\widehat{C}(g)$ we do not enforce a 'sum-to-one' constraint per mixture, as we expect cells in the bulks, for which we did not obtain a reference profile. Our correlation-based loss function yields deconvolution results that ensure cell type fold changes are conserved in the estimated proportions.
If cell type j in sample n has the proportion $C_{j,n}$ and sample n' has $C_{j,n'}$ with $C_{j,n'} = 2 * C_{j,n}$. Then, the fold change for cell type j is

$$\frac{C_{j,n}}{C_{j,n'}} = \frac{1}{2}.$$

Due to the correlation-based loss function, the estimated proportions show the same fold change

$$\frac{\widehat{C}_{j,n}}{\widehat{C}_{j,n'}} = \frac{1}{2}.$$

We advise users not to interpret the absolute size of $\widehat{C}$ nor to compare estimated cell proportions between cell types.

```
# print(model$pics$true_vs_esti[[1]])
trained.artificial.mixtures.pic <- ggplot_true_vs_esti(
  DTD.model = model,
  X.matrix = X.matrix,
  test.data = test.data,
  title = " trained model, test bulks",
  estimate.c.type = "direct",
)
print(trained.artificial.mixtures.pic[[1]])
```

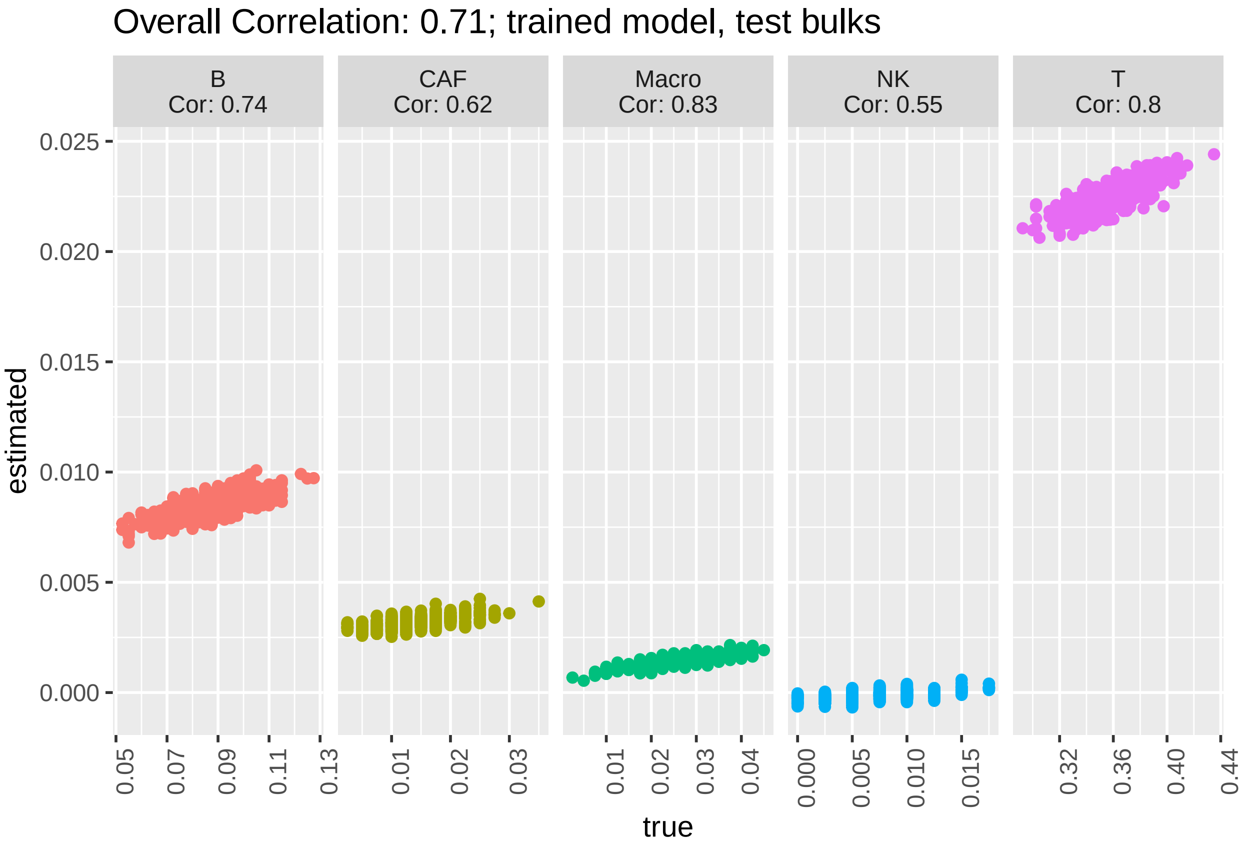

Deconvolute bulk profiles

A *DTD* model can be applied to estimate cellular compositions via the `estimate_c` function. As input, it takes a reference matrix X, the data to be deconvoluted (notice, a expression matrix, not a list), and the *DTD* model. The output is an estimated cellular composition matrix $\widehat{C}(g)$. Results can be visualized with the `ggplot_true_vs_esti` function. Pass, exemplary, the `bulk.list` to the `test.data` parameter. In the following plot, we included both test and training bulks in the plots. Therefore, the shown correlation is overoptimistic. For comparison, we show the deconvolution results using the baseline model on the bulk data again.

```
# estimate cellular compositions of bulk profiles:
estimated.c.bulk <- estimate_c(
  X.matrix = X.matrix,
  new.data = bulk.list$mixtures,
  DTD.model = model
)
# visualize true bulk C versus estimated bulk C:
trained.bulk.deconvolution.pic <- ggplot_true_vs_esti(
  DTD.model = model,
  X.matrix = X.matrix,
  test.data = bulk.list,
  title = " reconstructed bulks; trained model",
  estimate.c.type = "direct",
  shape.indi = ifelse(tumor.names %in% train.melanomas, "train", "test"),
  show.legend = TRUE
)

print(trained.bulk.deconvolution.pic[[1]])
print(untrained.bulk.deconvolution.pic[[1]])
```

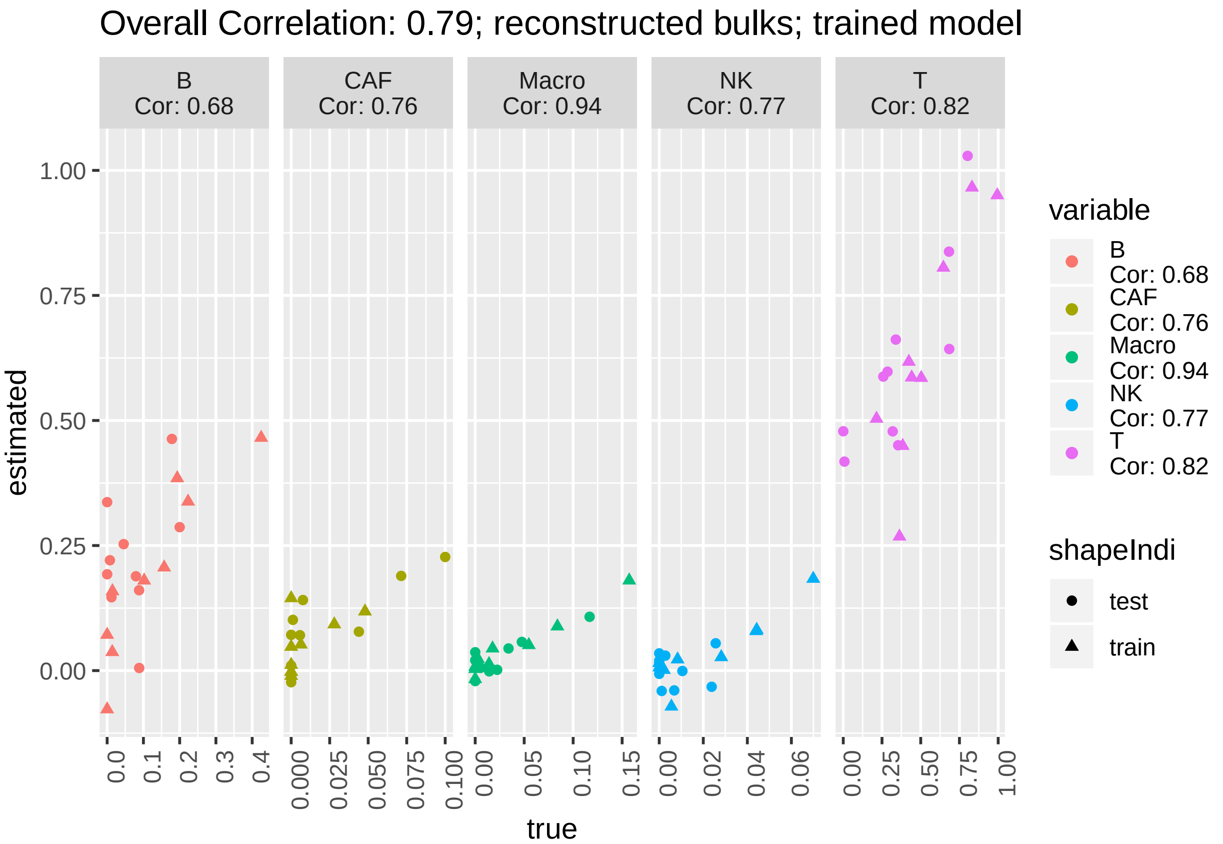
Overall Correlation: 0.79; reconstructed bulks; trained model
B
Cor: 0.68
CAF
Cor: 0.76
Macro
Cor: 0.94
NK
Cor: 0.77
T
Cor: 0.82
estimated
1.00
0.75
0.50
0.25
0.00
0.0
0.1
0.2
0.3
0.4
0.000
0.025
0.050
0.075
0.100
0.00
0.05
0.10
0.15
0.00
0.02
0.04
0.06
0.00
0.25
0.50
0.75
1.00
true
variable
B
Cor: 0.68
CAF
Cor: 0.76
Macro
Cor: 0.94
NK
Cor: 0.77
T
Cor: 0.82
shapeIndi
test
train

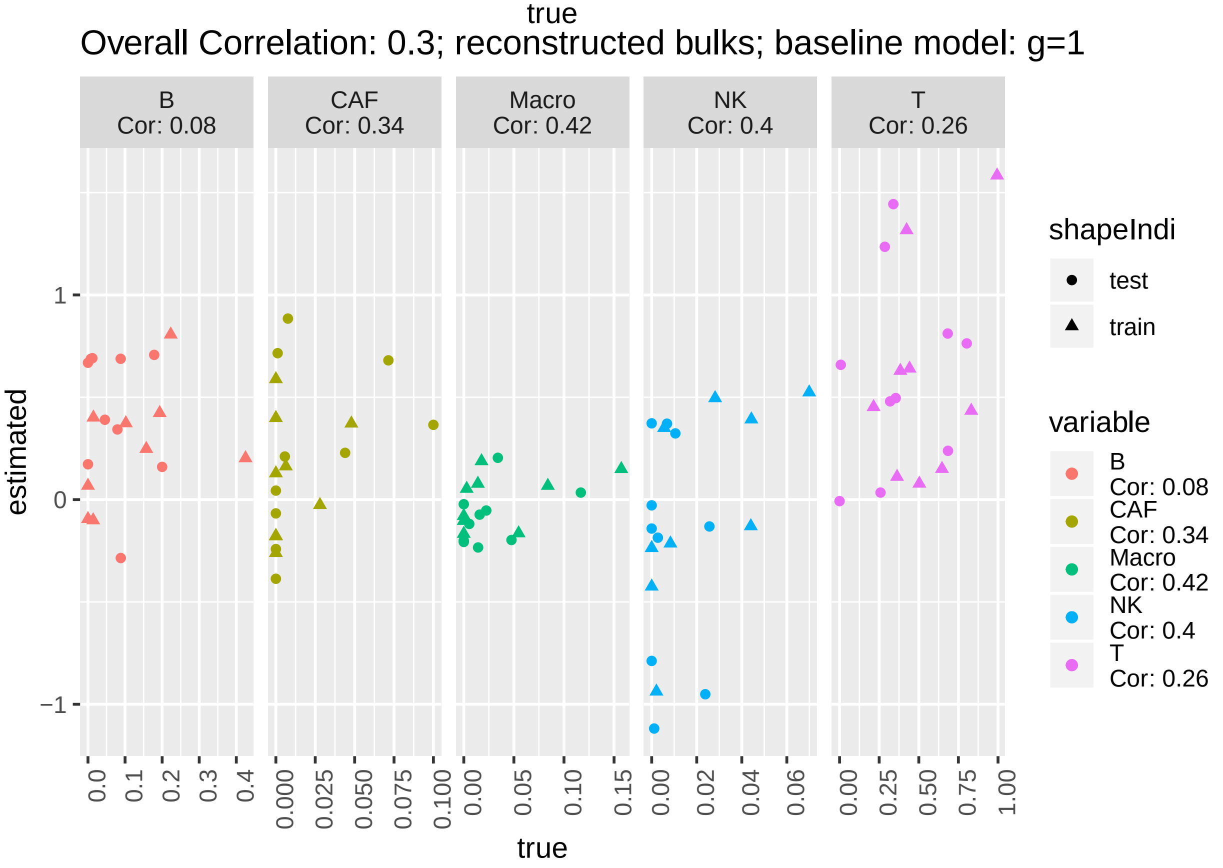
Overall Correlation: 0.3; reconstructed bulks; baseline model: g=1
B
Cor: 0.08
CAF
Cor: 0.34
Macro
Cor: 0.42
NK
Cor: 0.4
T
Cor: 0.26
estimated
1
0
−1
0.0
0.1
0.2
0.3
0.4
0.000
0.025
0.050
0.075
0.100
0.00
0.05
0.10
0.15
0.00
0.02
0.04
0.06
0.00
0.25
0.50
0.75
1.00
true
shapeIndi
test
train
variable
B
Cor: 0.08
CAF
Cor: 0.34
Macro
Cor: 0.42
NK
Cor: 0.4
T
Cor: 0.26

sessionInfo

```r
sessionInfo()
```

```
## R version 3.5.0 (2018-04-23)
## Platform: x86_64-apple-darwin15.6.0 (64-bit)
## Running under: macOS  10.15.1
##
## Matrix products: default
## BLAS: /System/Library/Frameworks/Accelerate.framework/Versions/A/Frameworks/vecLib.framework/Versions
## LAPACK: /Library/Frameworks/R.framework/Versions/3.5/Resources/lib/libRlapack.dylib
##
## locale:
## [1] en_US.UTF-8/en_US.UTF-8/en_US.UTF-8/C/en_US.UTF-8/en_US.UTF-8
##
## attached base packages:
## [1] parallel  stats     graphics  grDevices utils     datasets  methods
## [8] base
##
## other attached packages:
##  [1] tictoc_1.0          GEOquery_2.50.5     Biobase_2.42.0
##  [4] BiocGenerics_0.28.0 DTD_1.1             nnls_1.4
##  [7] reshape2_1.4.3      ggplot2_3.2.1       Matrix_1.2-17
## [10] matrixStats_0.55.0
##
## loaded via a namespace (and not attached):
##  [1] tidyselect_0.2.5 xfun_0.10        purrr_0.3.2      lattice_0.20-38
##  [5] colorspace_1.4-1 vctrs_0.2.0      htmltools_0.3.6  yaml_2.2.0
##  [9] rlang_0.4.2      pillar_1.4.2     glue_1.3.1       withr_2.1.2
## [13] tweenr_1.0.1     lifecycle_0.1.0  plyr_1.8.4       stringr_1.4.0
## [17] munsell_0.5.0    gtable_0.3.0     evaluate_0.14    labeling_0.3
## [21] knitr_1.25       curl_4.2         Rcpp_1.0.3       readr_1.3.1
## [25] scales_1.1.0     backports_1.1.5  limma_3.38.3     farver_2.0.1
## [29] ggforce_0.2.2    hms_0.5.1        digest_0.6.23    stringi_1.4.3
## [33] dplyr_0.8.3      polyclip_1.10-0  grid_3.5.0       tools_3.5.0
## [37] magrittr_1.5     lazyeval_0.2.2   tibble_2.1.3     crayon_1.3.4
## [41] tidyr_1.0.0      pkgconfig_2.0.3  zeallot_0.1.0    MASS_7.3-51.4
## [45] xml2_1.2.2       assertthat_0.2.1 rmarkdown_1.14   R6_2.4.1
## [49] compiler_3.5.0
```

References

Bates, Douglas, and Martin Maechler. 2019. *Matrix: Sparse and Dense Matrix Classes and Methods*. https://CRAN.R-project.org/package=Matrix.

Bengtsson, Henrik. 2019. *MatrixStats: Functions That Apply to Rows and Columns of Matrices (and to Vectors)*. https://CRAN.R-project.org/package=matrixStats.

Görtler, Franziska, Stefan Solbrig, Tilo Wettig, Peter J. Oefner, Rainer Spang, and Michael Altenbuchinger. 2018. *Research in Computational Molecular Biology: 22nd Annual International Conference, Recomb 2018, Paris, France, April 21-24, 2018, Proceedings (Lecture Notes in Computer Science)*. Springer.

Mullen, Katharine M., and Ivo H. M. van Stokkum. 2012. *Nnls: The Lawson-Hanson Algorithm for Non-Negative Least Squares (Nnls)*. https://CRAN.R-project.org/package=nnls.

Pedersen, Thomas Lin. 2019. *Ggforce: Accelerating 'Ggplot2'*. https://CRAN.R-project.org/package=ggforce.

R Core Team. 2018. *R: A Language and Environment for Statistical Computing*. Vienna, Austria: R Foundation for Statistical Computing. https://www.R-project.org/.

Tirosh, I., B. Izar, S. M. Prakadan, M. H. Wadsworth, D. Treacy, J. J. Trombetta, A. Rotem, et al. 2016. "Dissecting the Multicellular Ecosystem of Metastatic Melanoma by Single-Cell RNA-Seq." *Science* 352 (6282). American Association for the Advancement of Science (AAAS): 189–96. https://doi.org/10.1126/science.aad0501.

Wickham, Hadley. 2007. "Reshaping Data with the reshape Package." *Journal of Statistical Software* 21 (12): 1–20. http://www.jstatsoft.org/v21/i12/.

———. 2016. *Ggplot2: Elegant Graphics for Data Analysis*. Springer-Verlag New York. https://ggplot2.tidyverse.org.
